# Novel Combinatorial Regimen of Garcinol and Curcuminoids for Non-alcoholic Steatohepatitis (NASH) in Mice

**DOI:** 10.1038/s41598-020-64293-w

**Published:** 2020-05-04

**Authors:** Muhammed Majeed, Shaheen Majeed, Kalyanam Nagabhushanam, Lincy Lawrence, Lakshmi Mundkur

**Affiliations:** 1 Sami Labs Limited, Peenya Industrial Area, Bangalore, 560 058 Karnataka India; 2 Sabinsa Corporation, 750 Innovation Circle, Payson, UT 84651 USA; 3 Sabinsa Corporation, 20 Lake Drive, East, Windsor, NJ 08520 USA

**Keywords:** Biologics, Fat metabolism

## Abstract

Non-alcoholic steatohepatitis (NASH) is a progressive form of Non-alcoholic fatty liver disease (NAFLD), a chronic liver disease with a significant unmet clinical need. In this study, we examined the protective effects of *Garcinia indica* extract standardized to contain 20% w/w of Garcinol (GIE) and 95% Curcuminoids w/w from Curcuma longa (Curcuminoids) in a Stelic animal model (STAM) of NASH. The STAM mice developed steatosis, hepatocyte ballooning, and inflammation, which were significantly reduced by the combination of GIE and Curcuminoids, resulting in a lower NAFLD activity score. The treatment reduced fibrosis as observed by Sirius red staining, liver hydroxyproline content and mRNA levels of TGF- β and collagen in the liver. Immunostaining with alpha-smooth muscle actin (α SMA) revealed a significant reduction in hepatic stellate cells. Intriguingly, the combination regimen markedly decreased the mRNA levels of MCP1 and CRP and both mRNA and protein levels of TNF-α. NF-kB, reduced the hepatic and circulating FGF21 levels and altered the nonenzymatic (glutathione) and enzymatic antioxidant markers (Glutathione peroxidase, and superoxide dismutase). Our results suggest that the combination of GIE and Curcuminoids can reduce the severity of NASH by reducing steatosis, fibrosis, oxidative stress, and inflammation. The results suggest that the combinatorial regimen could be an effective supplement to prevent the progression of liver steatosis to inflammation and fibrosis in NASH.

## Introduction

Nonalcoholic fatty liver disease (NAFLD) is the most common cause of chronic liver disorders^1,2^. It includes the spectrum of liver disease, ranging from benign fatty liver to hepatocellular carcinoma. It is histologically further categorized into the nonalcoholic fatty liver (NAFL) and nonalcoholic steatohepatitis (NASH). NAFL, as the early-stage disease shows the presence of excessive fat in the liver (hepatic steatosis) with no evidence of hepatocellular injury, while NASH is characterized by the accumulation of fat accompanied by infiltration of inflammatory cells and cellular damage, which can progress to cirrhosis, liver failure, and liver cancer^3^. NASH is the hepatic manifestation of metabolic disorder and is closely associated with type 2 diabetes, obesity, insulin resistance anda systemic inflammatory state^4–8^. Consequently, NASH patients have a higher risk of cardiovascular events and neoplasia, resulting in a higher rate of mortality^9,10^. It is usually a silent disease with minimum symptoms, while weight loss, fatigue, and weakness develop as the disease progresses.

Although the disease development and progression are still not well elucidated, the most accepted theory to explain the pathogenesis is “multiple-hits hypothesis”. The initial hit leads to the development of simple steatosis, while liver cell inflammation and apoptosis are induced by secondary hits, leading to mitochondrial dysfunction, oxidative stress, lipid peroxidation, gut dysbiosis, and Kupffer cell activation, which finally results in NASH^11,12^. Presence of damaged hepatic cells, inflammation and fibrosis  characterize the disease^13,14^. Recent experiments have shown that disruption of endoplasmic reticulum (ER) homeostasis, or ER stress, induces both the development of steatosis and progression to NASH^15–17^. The induction of ER stress has been described in the livers of genetic, diet-induced and obese animal models of NASH and also in patients with NAFLD or NASH^16,18–21^.

NASH is a significant burden to the public health system, with no approved drugs for treatment. The primary mode of treatment is lifestyle management, while pioglitazone and Vitamin E have been used to reduce cellular injury, fibrosis and improve steatohepatitis^22^. Several novel medications, including agonists of peroxisome proliferator-activated receptor (PPAR)-alpha and PPAR-gamma and semisynthetic bile acid analogs, targeting different stages of the disease are in the pipeline^23^.

Curcumin C3 Complex is a proprietary commercial extract from the rhizomes of Curcuma longa, standardized for 95% curcuminoids. C3 Complex refers to the presence of three natural actives:Curcumin (75–81%), demethoxycurcumin(15–19%) and Bisdemethoxycurcumin (2.2–6.5%), collectively known as curcuminoids. It is the most extensively studied an﻿d clinically documented extractand is known to have a wide range of therapeutic actions, including antioxidant, anti-inflammatory, anticancer and lipid regulatory activities *in vitro* and animal models^24^. The hepatoprotective activity of Curcuminoids is reported to be mediated by the reduction of oxidative stress and attenuation of nuclear factor kappa B (NF-κB) mediated anti-inflammatory activity^25–28^. Garcinol, a polyisoprenylatedbenzophenone isolated from the fruit rinds of *Garcinia indica*, is known to have antioxidant, anti-glycation, anti-cancer, and protective action against drug-induced liver damage^29–32^. We hypothesized that the combination of *Garcinia indica* extract containing 20% Garcinol (GIE) andCurcuminoids would act on different pathways of NASH pathogenesis and have synergistic protective activity. We used the STAM mouse model of NASH to study the hepatoprotective effect of GIE and Curcuminoids individually and in combination.

The STAM mouse model developed by Fuji *et al*. shows the progressive development of NASH, fibrosis and finally, hepatocellular carcinoma^33^. This pathological progression seen in the mouse model is very similar to the human disease, in the rapid and stepwise progression from steatosis to NASH to fibrosis. In this study, we show the beneficial effects of GIE, Curcuminoids and their combination in alleviating the progression of NASH in the STAM mice model.

## Method

### Streptozotocin and HFD induced STAM™ model

All methods using animals were carried out as per the relevant guidelines and regulations with approval from the animal ethics committee of SMC laboratories Japan (IACUC). The *in vivo* studies were conducted as per theAnimal Welfare Assurance for foreign institutions from the Office of Laboratory Animal Welfare (Animal Welfare Assurance number: A5037–01). C57BL/6 (14-day-pregnant female mice) were obtained from Japan SLC, Inc. (Japan). The animals were housed and cared for by following the Japanese Pharmacological Society Guidelines for Animal Use [Act on Welfare and Management of Animals, Ministry of the Environment, Act No. 105 of October 1, 1973, Standards Relating to the Care and Management of Laboratory Animals and Relief of Pain (Notice No.88 of the Ministry of the Environment, April 28, 2006) and Guidelines for Proper Conduct of Animal Experiments (Science Council of Japan, June 1, 2006)]. The animals were maintained in an SPF facility under controlled conditions of temperature (23 ± 2 °C), humidity (45 ± 10%), lighting (12-hour artificial light and dark cycles; light from 8:00to 20:00) and air exchange. High pressure was maintained in the experimental room to prevent contamination of the facility. NASH was established in male mice by a single subcutaneous injection of 200 µg streptozotocin (Sigma, USA) 2 days after birth and feeding with a high-fat diet (CLEA Japan, Japan) *ad libitum* from 4 weeks of age (age 28 ± 2 days)^33^. The viability, clinical signs (lethargy, twitching, labored breathing) and behavior were monitored daily. Mice were observed for significant clinical signs of toxicity, morbidity and mortality before administration, just after administration and 1 hour after administration. At the termination of each study, animals were sacrificed by exsanguination through direct cardiac puncture under isofluraneanesthesia (Pfizer Inc.) and Liver and blood samples were collected for histopathology and biomarkers analysis.

### Test materials and experimental design

**Figure 1:**
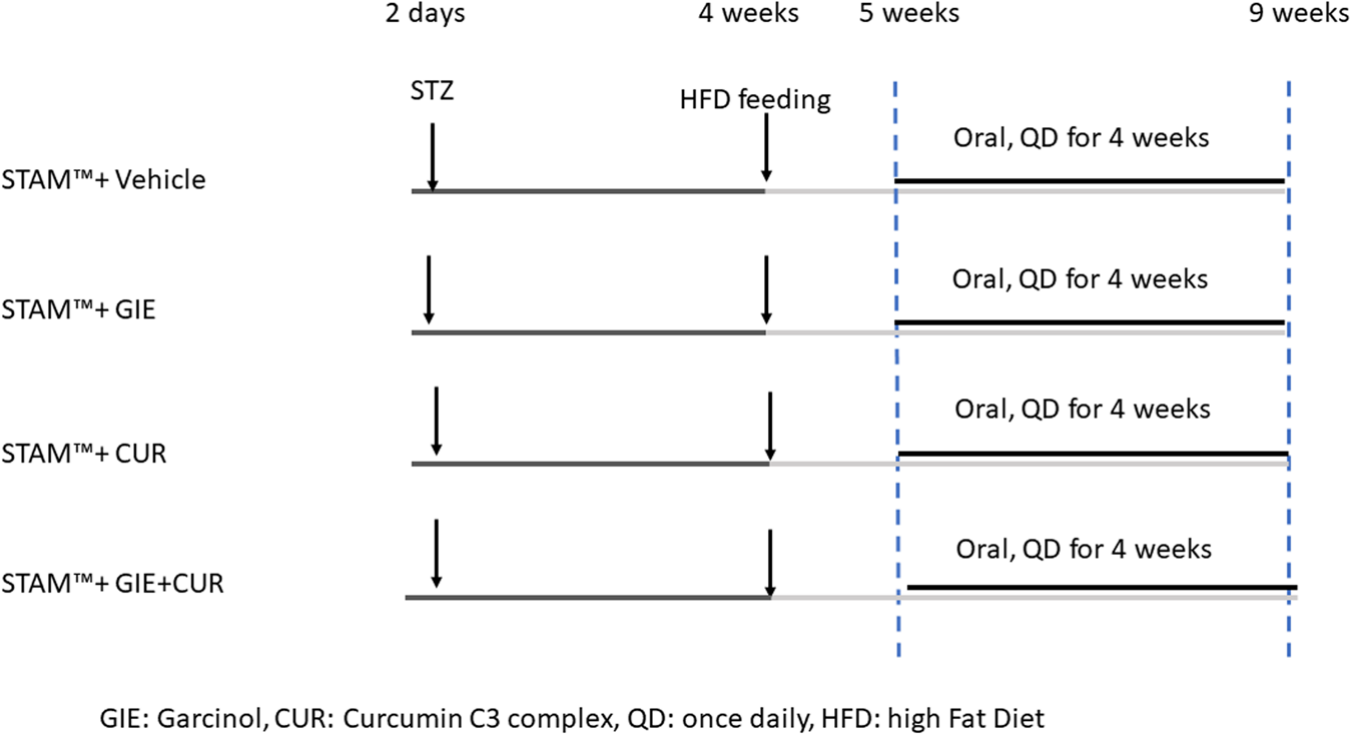
Experimental design:STAM mice were generated as described in the methods section. High fat diet feeding was started at 4 weeks of age and continued until 9 weeks. The treatment regimen was started from week 5 and the animals were orally dosed with vehicle*, Garcinia indica* extract GIE), Curcuminoids (CUR) and their combination (GIE + CUR) once daily for 4 weeks. The mice were humanely sacrificed at the end of 9 weeks for further evaluation of the effects.

GIE (LIVINOL) and Curcuminoids (Curcumin C3 Complex) were from Sabinsa Corporation. GIE was standardized to contain 20% w/w Garcinol, while Curcuminoids, is a proprietary commercial extract from the rhizomes of Curcuma longa, standardized for 95% w/w total curcuminoids (Curcumin (75–81%), demethoxycurcumin (15–19%) and Bisdemethoxycurcumin (2.2–6.5%). Garcinol was extracted from *Garcinia indica* and diluted to 20% w/w with microcrystalline cellulose powder to get 20% w/w of Garcinol in GIE. Both samples(dry powders) were weighed and suspended in the vehicle [0.5% methyl cellulose]. STAM mice were divided into four groups (N = 8) in each group at the age of 5weeks,two days before the start of treatment. The animals were orally administered with the test material or vehicle in a volume of 5 mL/kg body weight (BW) once daily for four weeks, starting from week 5 to week 9. The control animals received vehicle (0.5% methylcellulose), the second group of animals received GIE at a dose of 10 mg/kg BW, the third group received Curcuminoids at a dose of 50 mg/kg BW, while the fourth group of animals was given a combination of GIE (10 mg/kg BW) and Curcuminoids(50 mg/kg BW). The experimental design is shown in Fig. 1

### Sample collection

For plasma samples, non-fasting blood was collected in polypropylene tubes with anticoagulant (Novo-Heparin), centrifuged at 1,000 × g for 15 min at 4 °C and the supernatant was collected and stored at −80 °C. The left medial lobe and the caudate lobe of the liver were snap-frozen in liquid nitrogen and stored at −80 °C. For paraffin-embedded liver blocks, the left lateral lobe was collected and cut into 6 pieces. Two pieces of left lateral lobe were fixed in Bouin’s solution and then embedded in paraffin and samples were stored at room temperature.

## Biochemical Measurements

Non-fasting blood glucose was measured by using Stat Strip glucose meter (NIPRO CORPORATION, Japan). For plasma biochemistry, non-fasting blood was collected in polypropylene tubes with anticoagulant (Novo-Heparin, Mochida Pharmaceutical Co. Ltd., Japan) and centrifuged at 1,000 g for 15 minutes at 4 °C. The supernatant was collected and stored at−80 °C until use. Plasma ALT and total cholesterol were measured by FUJI DRI-CHEM 7000 (Fujifilm, Japan). Plasma AST and albumin were measured by commercial kits (Prietest™, Robonik®, India). Lipid peroxidation was estimated from the plasma level of malondialdehyde (MDA) as described by Yoshioka et al., 1979. Briefly, 0.2 ml aliquot of plasma was shaken with 1 ml of 20% trichloroacetic acid (TCA). To the mixture, 0.4 ml of 0.67% thiobarbituric acid (TBA) was added and warmed for 30 minutes in a boiling water bath followed by rapid cooling. Then 0.8 ml of n-butyl-alcohol was added and shaken; the mixture was centrifuged at 3,000 rpm for 10 minutes. The resultant n-butyl-alcohol layer was taken into a separate tube and MDA content in the plasma was determined from the absorbance at 535 nm^34^.

### Measurement of liver triglyceride content

Liver total lipid were measured by Folch’s method^35^. Liver samples were homogenized in chloroform-methanol (2:1, v/v) and incubated overnight at room temperature. After washing with chloroform-methanol-water (8:4:3, v/v/v), the extracts were evaporated to dryness and dissolved in isopropanol. Liver triglyceride content was measured by Triglyceride E-test (Wako Pure Chemical Industries, Ltd., Japan).

### Measurement of liver hydroxyproline content

Frozen liver samples were processed by an alkaline-acid hydrolysis method to quantify the liver hydroxyproline content. The defatted liver samples were dried in the air, dissolved in 2 N NaOH at 65 °C, and autoclaved at 121 °C for 20 minutes. The lysed samples (400 µL) were acid-hydrolyzed and neutralized. Acetate citrate buffer was added to the samples, followed by centrifugation to collect the supernatant. A standard curve of hydroxyproline was constructed with serial dilutions of trans-4-hydroxy-L-proline (Sigma-Aldrich) starting at 16 µg/mL. The prepared samples and standards were mixed with equal volume of chloramine T solution (Wako Pure Chemical Industries) and incubated for 25 minutes at room temperature. The samples were then mixed with Ehrlich’s solution (400 µL) and heated at 65 °C for 20 minutes to develop the color and the optical density of each supernatant was measured at 560 nm. The concentrations of hydroxyproline were calculated from thestandard curve. Protein concentrations of liver samples were determined using a BCA protein assay kit (Thermo Fisher Scientific, USA) and used to normalize the calculated hydroxyproline values and expressed as µg per mg protein.

## Liver antioxidant measurements

### MDA

Malondialdehyde, a lipid peroxidation end product in tissue homogenate, was determined according to the method of Beuge and Aust., 1978 with some modifications^36^. The tissue homogenate was mixed with an equal volume of TBA-TCA-HCl solution (0.5% TBA, 20% TCA and 0.25 N HCl). The mixture was heated for 30 min in a boiling water bath (95–100 °C) and cooled immediately. The tubes were centrifuged at 10,000 rpm for 10 min and absorbance of the supernatant was read at 532 nm. The level of lipid peroxides was expressed as µM MDA formed/mg protein.

### SOD

The activity of SOD was measured by WST-1 method using a kit as per the manufacturer’s instructions (Elabsciences). Xanthine Oxidase (XO) catalyzes the reaction of WST-1 with O_2_^−^ to generate a water-soluble formazan dye. SOD catalyzes the disproportionation of superoxide anions. The activity of SOD is negatively correlated with the amount of formazan dye.

### Glutathione peroxidase (GPx)

Glutathione peroxidase activity was determined according to the method of Hafeman, *et al*.^37^. Glutathione peroxidase degrades H_2_O_2_ in the presence of glutathione (GSH), thereby depleting it. The remaining GSH is measured using Ellman’s reagent (5,5′-dithio-bis(2-nitrobenzoic acid) (DTNB), which gives a colored complex. The enzyme activity was expressed as units/mg protein.

### Glutathione (GSH) content

Reduced glutathione was determined based on the method of Moron, *et al*.^38^. GSH is measured by its reaction with DTNB to give a yellow colored complex with maximum absorption at 412 nm A standard graph was prepared with different concentrations (62.5–1000 μM) of GSH. The GSH content in the sample was calculated from the standard graph and expressed as μmol/mg protein.

### ELISA

The concentration of TNF-α, adiponectin and FGF21 in plasma andliver homogenate were carried out by ELISA (R&D Systems (Minneapolis, Minnesota, USA) as per the manufacturer’s instructions. The results were expressed as concentration per mL for plasma and per mg protein for liver homogenate.

### RNA extraction and quantitative RT-PCR

For cDNA samples, the other 2 pieces of left lateral lobe were snap-frozen in liquid nitrogen and stored at −80 °C until use. Total RNA was extracted from liver samples using RNAiso (Takara Bio, Japan) according to the manufacturer’s instructions. One μg of RNA was reverse-transcribed using MMLV-RT (Invitrogen). The samples were frozen in liquid nitrogen and stored at −80 °C until use. Quantitative real-time PCR (qRT-PCR) was performed with SYBR Green I fluorescent dye using Light cycler 96 according to the manufacturer’s instructions (Light Cycler^®^FastStart DNA Master SYBR Green I, Roche). The primers used for the analysis are provided in Supplementary Table 1. Expression levels for all genes were normalized to β-actin gene amplification. The gene expression of the target gene in each test sample was determined by relative quantification using the comparative Ct (ΔΔCt) method.

### Histological analyses

For Hematoxylinand Eosin staining, sections were cut from paraffin blocks of liver tissue prefixed in Bouin’ssolution and stained with Lillie-Mayer’s Hematoxylin (Muto Pure Chemicals Co., Ltd., Japan) and eosin solution (Wako Pure Chemical Industries). NAFLD Activity Score (NAS) was calculated according to the criteria of Kleiner^39^. To visualize collagen deposition, Bouin’s fixed liver sections were stained using a picrosirius red solution (Waldeck, Germany). For quantitative analysis of fibrosis area, bright field images of Sirius red-stained sections were captured around the central vein using a digital camera (DFC295; Leica, Germany) at 200-fold magnification, and the positive areas in 5 fields/section were measured using ImageJ software (National Institute of Health, USA).

### Immunohistochemical analysis

The tissues were deparaffinized using xylene wash and antigens were retrieved by the microwave method as described earlier. Endogenous peroxidase activity was blocked by treating the tissues with 3.0% hydrogen peroxide for 10 minutes. The sections were washed in distilled water two times for 5 minutes. The tissues were permeabilized with 0.04% Triton X-100 in TBS-T for 10 minutes and nonspecific binding was blocked by incubating with 10% serum in TBS-T at room temperature. Primary antibody (αSMA- Abcam) staining was carried out for 2 hours at room temperature followed by overnight incubation at 4 °C, while the second antibody was incubated at room temperature for one hour. The sections were visualized by incubating with Diaminobenzidine (DAB) solution (0.5 mg/ml containing 0.015% hydrogen peroxide) in dark for 5 minutes at room temperature, washed and counterstained with hematoxylin. Images were captured using the Brightfield microscope (Nikon Eclipse)

### Immunoblotting

Frozen liver from the animals were homogenized and the cells were lysed using ice-cold RIPA buffer containing protease (1x protease inhibitor cocktail – HI media) and phosphatase (sodium orthovanadate 1 mM). Protein concentration was estimated by the Bradford method **(**Sigma, USA). Cellular protein (100 μg) was loaded per lane in denatured 10% polyacrylamide gel (SDS-PAGE). The separated proteins were transferred to a polyvinylidenedifluoride membrane (Invitrolon™ PVDF, Thermo Fisher Scientific, USA) and blocked in 5% nonfat dry milk for 2 hours. Membranes were then incubated with the appropriate dilutions of anti-mouse primary antibodies at 4 °C for 18 hours, followed by horseradish peroxidase-conjugated secondary antibody (Thermo Scientific, USA) for 2 hours at 37 °C. The details of the antibodies are provided in Supplementary Table 2. Immunoreactive protein bands were detected by ECL ((Pierce ECL plus,Thermo Scientific, USA). Immunoblots were quantified using Image J software (version 1.52a, National Institute of Health, USA).

### *In vitro* evaluation of steatosis and inflammation

HepG2 hepatocytes (American Type Culture Collection (ATCC, Rockville, MD, USA) were used to study the steatosis  due to their sensitivity to lipid accumulation^40^. The cells were grown in DMEM (Gibco, NY, USA) supplemented with 10% fetal bovine serum (FBS) (Thermo Fisher Scientific, USA), and plated at a density of 2 × 10^4^ cells/well in two 96-well plate or 2.5 × 10^5^ cells in a 6 well plate and allowed to grow until they were 70% confluent andpre-treated with different concentrations of the GIE, Curcuminoids or their combination for 24 hours. Steatosis was induced by replacing the medium with serum free media containing 1% fatty free BSA and 1 mM FFA [oleic acid (0.8 mM) and palmitic acid (0.2 mM) in along with different concentrations of sample for another 24 hours. One plate was processed for cytotoxicity by MTT and the other for lipid staining using Oil-Red-O (Sigma-Aldrich, USA). Briefly, the cells were stained with ORO working solution [0.3%] for 60 min at room temperature after fixing with 10% formalin. The lipids stained with ORO were extracted from the cells using isopropanol and quantified at a wavelength of 520 nm. The treated cells were also processed for RNA and western blot analysis as described in earlier sections

Anti-inflammatory activity was evaluated in THP1 human monocytes (NCCS, Pune, India). ThP1 cells were cultured in X-VIVO™20 (BioWhittaker^™,^ MD, USA)serum free media stimulated with LPS (Sigma Chemicals, USA, 100 ng/mL) in the presence of GIE, Curcuminoids or their combination for 24 hours. The supernatants from THP1 were analyzed for inflammatory cytokines using Cytometric bead array in FACS celesta flow cytometer (CBA, BD biosciences, CA, USA).

### Statistical tests

Statistical analyses were performed using one-way ANOVA with Bonferroni Multiple Comparison Test using GraphPad Prism 6 (GraphPad Software Inc., USANonparametric Mann–Whitney test was used to compare the vehicle with the treated groups. *P* values < 0.05 were considered statistically significant. Results were expressed as mean ± SD.

## Results

### Mouse model

STAM mice are a well-defined model that enables monitoring of the natural progression of liver degeneration from fatty liver to NASH and fibrosis in a reasonably controlled manner. The animals were sacrificed at 9 weeks, at the stage of progression to fibrosis from NASH.

The mean body weight in all groups gradually increased during the treatment period and the animals weighed 20 g to 25 g after 14 weeks of age as this model is not obesity mice model and constitutively shows lower body weights compared to normal mice. None of the animals showed decline in general condition, suggesting that GIE and Curcuminoids did not produce any distinct toxicity at the selected doses.

### Serum biochemistry and body weight

The details of body and liver weight, serum biomarkers and liver enzyme levels are given in Table 1. No significant differences were observed in the mean body weight and the liver to body weight ratio between vehicle and treated groups. There were no significant differences in whole blood glucose levels between the vehicle and the other treatment groups. Furthermore, no significant changes in the plasma ALT, cholesterol and triglyceride levels were observed between the treated and control groups, while AST levels were lower in the GIE + Curcuminoid treated group (Table 1).

**Table 1:** Body Weight, Liver Weight and Biochemistry.

Parameter (Mean ± SD)	Vehicle	GIE	Curcuminoids	GIE + Curcuminoids
Body weight (g)	20.2 ± 1.3	20.3 ± 1.5	20.9 ± 1.7	20.0 ± 2.8
Liver weight (mg)	1480 ± 140	1467 ± 120	1584 ± 117	1415 ± 183
Liver-to-body weight ratio (%)	7.4 ± 0.7	7.3 ± 0.8	7.6 ± 0.9	7.1 ± 0.4
Whole blood glucose (mg/dL)	533 ± 75	564 ± 51	550 ± 43	605 ± 73
Plasma ALT (U/L)	51 ± 12	56 ± 12	56 ± 16	47 ± 14
Plasma AST (U/L)	123.7 ± 19.6	157 ± 25.4	125 ± 24.6	81.53 ± 11.2
Plasma total cholesterol (mg/dL)	152 ± 25	151 ± 18	162 ± 32	172 ± 50
Liver triglyceride (mg/g Liver)	57.5 ± 28.9	71.8 ± 33.3	93 ± 27.3	76.9 ± 29.8

Plasma level of biochemical markers were analyzed as described in the methods. No significant difference was observed in any of the parameters. ALT: alanine aminotransferase, AST: aspartate aminotransferase.

### Liver histopathology, and NAFLD activity score

**Figure 2:**
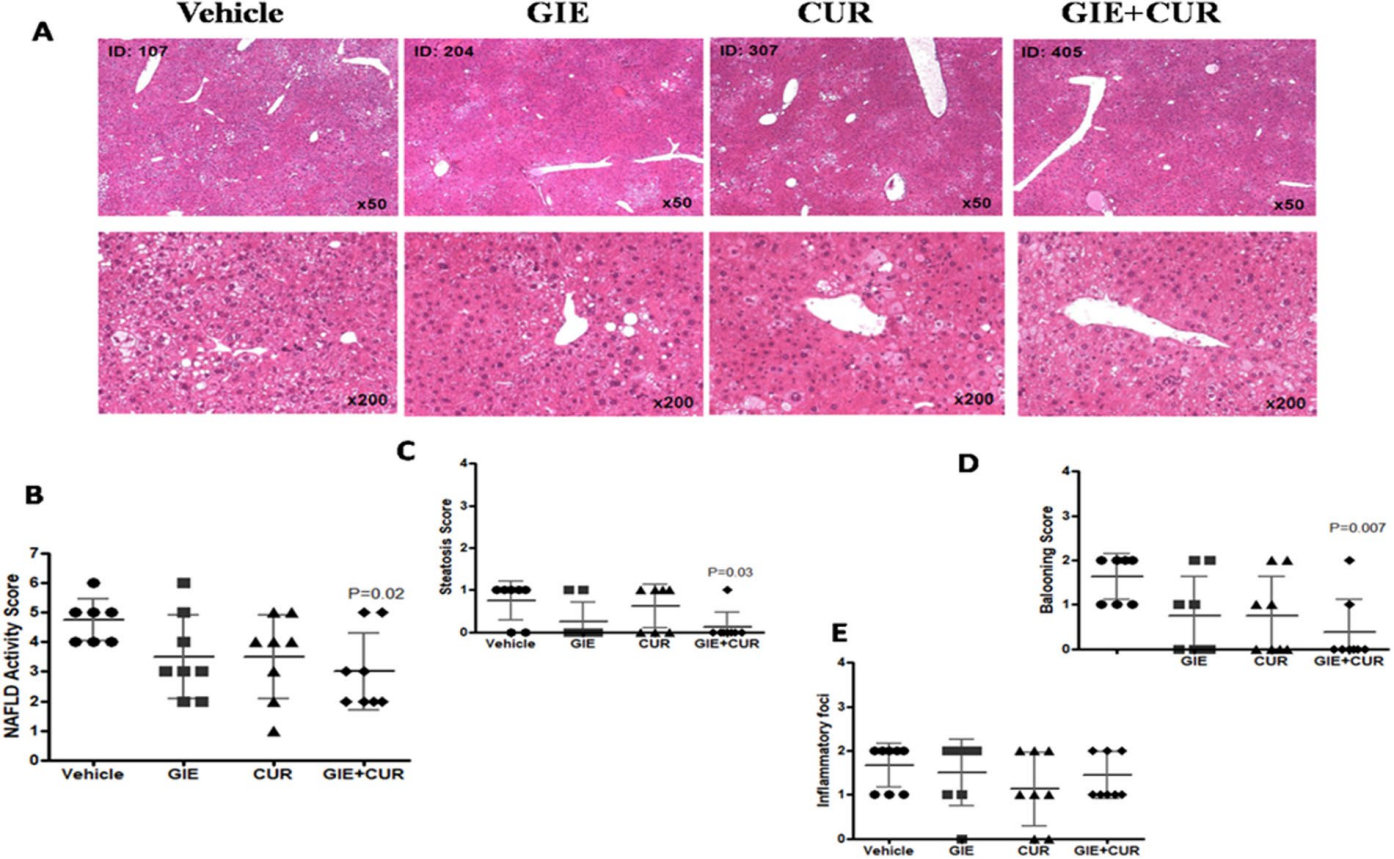
Reduction in NAS in STAM mice following GIE and Curcuminoids treatment: A:Representative H&E stained (50x and 200×), liver sections from the vehicle, *Garcinia indica* extract GIE), Curcuminoids (CUR) and their combination (GIE + CUR) treated NASH STAM mice collected at week 9. B: NAFLD activity score (NAS). C: steatosis score D: hepatocellular ballooning score E: lobular inflammation. N = 8 in each group.

The liver sections stained with hematoxylin and eosin (H&E) and NAFLD activity score are shown in Fig. 2 and Supporting Table S2, respectively. Liver sections from the vehicle group exhibited micro- and macro vesicular fat deposition, hepatocellular ballooning and inflammatory cell infiltration (Fig. 2A). Consistent with these observations, the NAFLD activity score (NAS) was 4.8 ± 0.7 in the vehicle. The NAS score reduced to 3.0 ± 1.3 in the GIE + Curcuminoids combination group (Fig. 2B). The decrease of NAS was not statistically significant in the individual group, while the combination was effective in reducing NAS by 37.5%, which was statistically significant (P = 0.02). Details of NAS enumeration are given in Supplementary Table 3. GIE showed a better effect in reducing Steatosis scores compared to curcuminoids, but the statistically significant change was observed only in the combination group (P = 0.03). Hepatocyte ballooning could not be detected in 50% of animals treated individually with GIE and Curcuminoids, while 75% of animals in the combination group did not develop hepatocyte ballooning (P = 0.007) (Fig. 2C,D). Inflammatory foci were reduced in the treated groups compared to the vehicle with Curcuminoids being most effective, but none of them showed a statistically significant effect (Fig. 2E).

### Effect of GIE and curcuminoids on liver fibrosis

**Figure 3:**
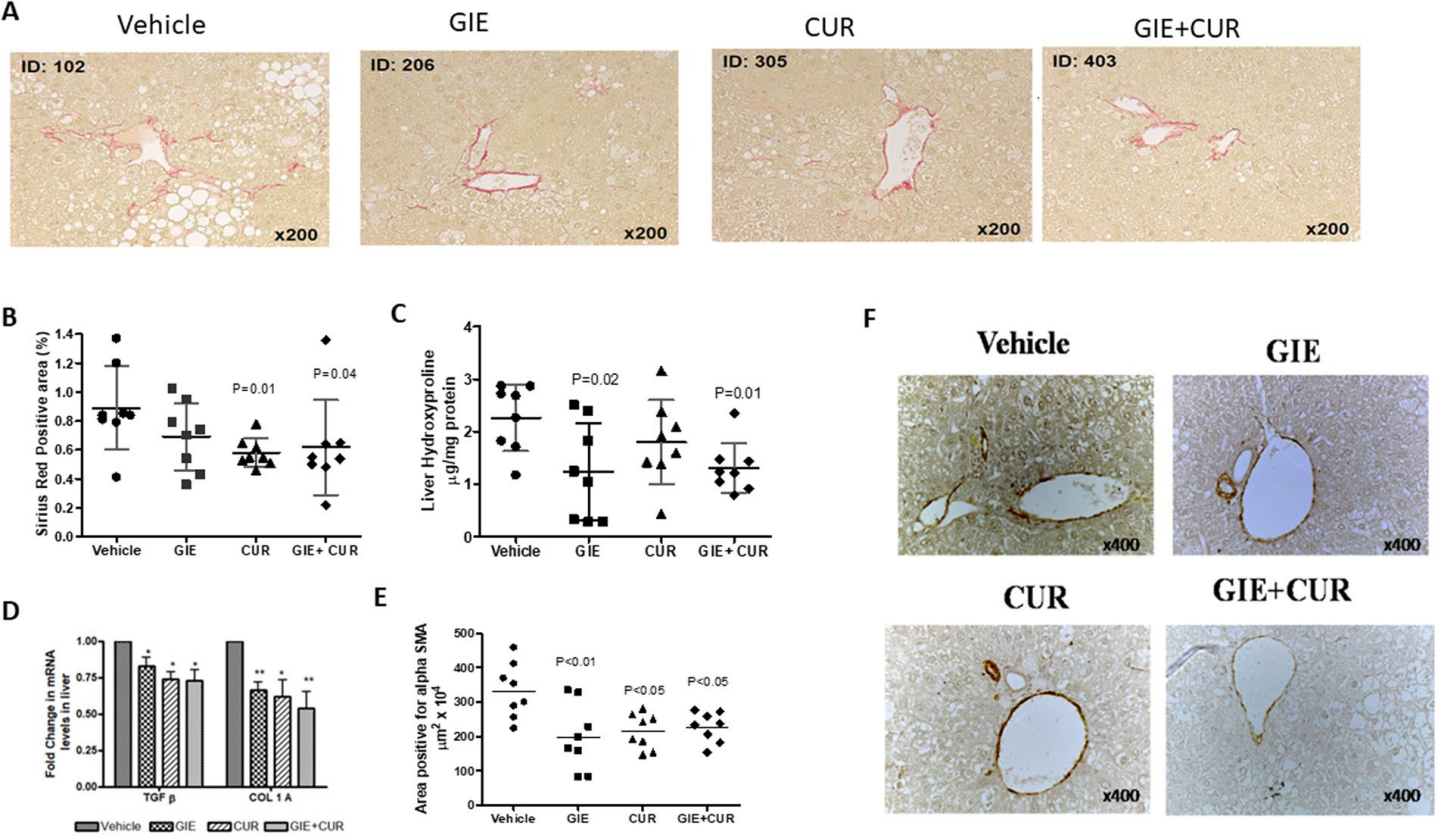
Reduction in markers related to Fibrosis following GIE andCurcuminoids treatment. A: RepresentativePicrosirius stained (200×), liver sections from vehicle, *Garcinia indica* extract (GIE), Curcuminoids(CUR) and their combination (GIE + CUR) treated NASH STAM mice collected at week 9. B:Fibrosis score C: Liver hydroxyproline concentration, and D: Relative mRNA levels of TGF-β and collagen 1 in the treated groups in comparison to Vehicle. E and F Representative Immunohistochemical staining (100×), liver sections with anti αSMA antibody and its quantification N = 8 in each group. * p < 0.05.

Representative photomicrographs of Sirius red-stained liver sections are shown in Fig. 3A. Liver sections from the vehicle group showed collagen deposition in the pericentral region of the liver lobule. The fibrosis area (Sirius red-positive area) was reduced by 22.47% with GIE, 34.83% with Curcuminoids and 30.33% with the combination compared with the vehicle group (Fig. 3A,B). In substantiation with these results, liver hydroxyproline concentrations were lower in the treated groups compared with the Vehicle (Fig. 3C) and the mRNA transcripts of collagen 1 and TGF-β were lower in the liver in comparison with the vehicle as assessed by quantitative real-time PCR. The expression of TGF-β was downregulated by 1.2, 1.35 and 1.37 folds while that of collagen 1 was downregulated by 1.5, 1.62 and 1.85-fold for GIE, Curcuminoids and the combination respectively (Fig. 3D). Myofibroblast plays a critical role in fibrogenesis which is characterized by the expression of α-smooth muscle actin (α-SMA). As shown in Fig. 3F, GIE, Curcuminoids and their combination significantly decreased the positive area for α-SMA in liver tissues as compared to the vehicle group. The collective outcome of the results as mentioned above suggests that the combination regimen exhibits a stronger anti-fibrotic effect.

### Effect of GIE and curcuminoids on inflammatory markers

**Figure 4:**
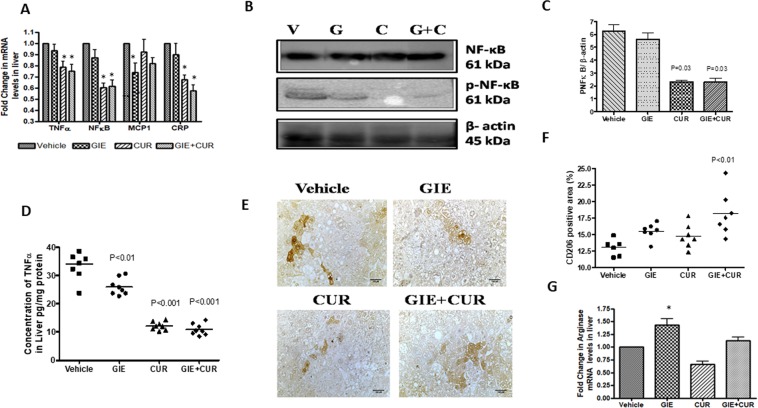
Reduction in inflammatory markers in STAM mice following GIE and Curcuminoids treatment. A: Relative mRNA levels of TNF-α NFkB, MCP1 and CRP in the treated groups in comparison to Vehicle, B: Immunoblot analysis of NFkB totaland phosphorylated protein levels in the liver homogenate of vehicle (V), GIE (G), CUR(C) and the combination (G + C),C: Quantitative analysis of p NFkB levels from the immunoblots (full-length blots are presented in Supplementary Figure 1. D:Concentration of TNF-α in liver homogenate E and F Representative Immunohistochemical staining of liver sections with anti CD206 antibody, G: Relative mRNA levels of arginase 1 in the liver of treated groups in comparison to Vehicle. *Garcinia indica* extract (GIE), Curcuminoids (Cur) and their combination (GIE + Cur) treated NASH STAM mice N = 8 in each group. *p < 0.05.

Increased hepatic inflammation and fibrosis are hallmarks of NASH progression. Accordingly, we sought to examine the effect of GIE, Curcuminoids and their combination on mRNA levels of inflammatory markers in liver tissues by qRTPCR (Fig. 4A). The mRNA transcripts of the inflammatory markers (TNF-α, NF-kB, and CRP) were downregulated by Curcuminoids (1.37, 1.65 and 1.48 folds respectively) and the combination of GIE + curcuminoids (1.41, 1.62 and 1.73 folds respectively) to a relatively greater extent compared to GIE (1.14,1.22 and 1.15 respectively). GIE lowered the levels of MCP1 transcript by 1.43 folds while it was only 1.1-fold and 1.28 folds for Curcuminoids and the combination, respectively. The phosphorylated NF-kB protein levels were also lower in Curcuminoids treated liver, suggesting its effect on controlling inflammation(Fig. 4B,C). Further, Curcuminoids and the GIE + Curcuminoids combination treatment significantly reduced the TNF-α protein concentration in the liver as compared to the vehicle group (Fig. 4D). Since GIE showed inhibition of MCP1, we assessed the M2 macrophage population in the liver by immunostaining with anti CD206 antibodies (Fig. 4E,F). Interestingly M2 macrophages were higher in the treated animals, especially in the combination while the mRNA level of Arginase1 was higher in GIE and the combination but not with Curcuminoids treatment (Fig. 4G).

**Figure 5:**
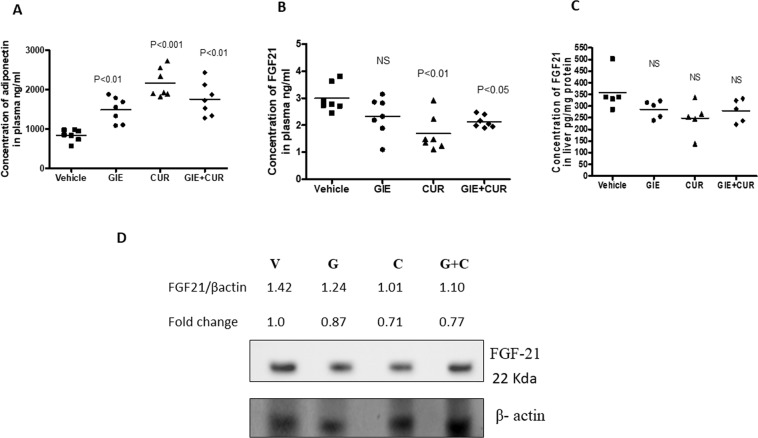
Changes in FGF21 and Adiponectin in STAM mice following GIE and Curcuminoids treatment: (**A**) Concentration of Adiponectin in plasma, (**B,C**) Concentration of FGF21 in plasma (**B**) and Liver (**C**). (**D**) Immunoblot analysis of FGF21 total protein levels in the liver homogenate of vehicle (V), GIE (G), CUR(C) and the combination (G + C), and thequantitative analysis.*Garcinia indica* extract (GIE), Curcuminoids (CUR) and their combination (GIE + CUR) treated NASH STAM mice.

The concentration of plasma adiponectin, an anti-inflammatory protein showed a significant increase in the GIE, Curcuminoids, and combination group, with a greater increase in animals treated with Curcuminoid alone (Fig. 5A). Serum FGF21 levels and hepatic mRNA of FGF21 are known to be elevated in human and mice models of NASH, due to FGF21 resistance^41,42^. The mice treated with curcuminoids and the combination of GIE and curcuminoids showed a decrease in FGF21 levels in serum and liver compared to the untreated control, while GIE had no impact on FGF21 levels (Fig. 5B,C). The immunoblot also showed a relatively lower expression of FGF21 protein in the liver of treated animals compared to untreated controls (Fig. 5D).

### Effect on MDA and antioxidant enzymes in serum and liver

**Figure 6:**
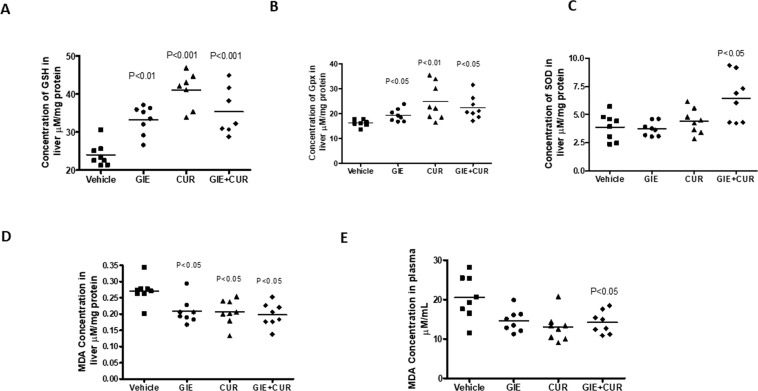
Increase in antioxidant enzymes and reduction in Oxidative stress in STAM mice following Garcinol and Curcuminoids treatment. (**A**) Concentration of GSH (glutathione). (**B**) Concentration of GPx (glutathione peroxidise) in liver homogenate (**C**) Concentration of SOD (superoxide dismutase). (**D**) MDA (Malondialdehyde) in liver homogenate, E:Concentration of MDA in plasma. N = 8 in each group.

The results of antioxidant levels in Garcinol, C3, and combination-treated NASH was shown in Fig. 6. GIE and combination regimen showed an increase in the levels of nonenzymatic antioxidants such as GSH and antioxidant enzyme GPx. The SOD activity was not influenced by GIE and Curcuminoids individually but was significantly increased by the combination(Fig. 6A–C). Malondialdehyde (MDA) is an indicator of lipid peroxidation, activates the inflammatory response and, consequently, causes cellular damage. As shown in Fig. 6D,E, GIE, Curcuminoids and combination treatment significantly reduced the MDA activity as compared to the vehicle group.

### *In vitro* evaluation of steatosis and inflammation

**Figure 7:**
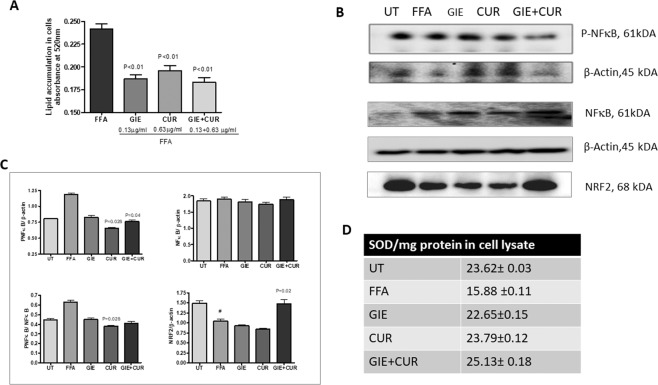
*In vitro* evaluation of steatosis and inflammation. FFA [oleic acid (0.8 mM) and palmitic acid (0.2 mM) were added to Human Hepatocyte cell line HepG2 for 24 hours in the presence of GIE, Curcuminoids and their combination. (**A**) The intracellular lipids were stained by Oil Red O (ORO) and quantified by absorbance at 525 nm. (**B**) Immunoblot analysis of hepatocyte cell lysate exposed to FFA in the presence of GIE (0.125 µg/ml), CUR(0.625 µg/ml) and the combination contained (0.125 µg/ml of GIE + 0.625 µg/ml of CUR). (**C**) Quantification of the immunoblot. (**D**) Concentration of SOD in the cell homogenate. *Garcinia indica* extract (GIE), Curcuminoids (CUR) and their combination (GIE + CUR), FFA: free fatty acids, UT: untreated.

To understand the mechanism of action of GIE and Curcuminoids, we explored their action on cultured hepatocytes *in vitro*. Since the animal experiments were carried outat 1:5 ratio of GIE and CUR, we initially carried out a cytotoxicity assay with different combinations of the two compounds and found that 0.125 µg/ml of GIE and 0.625 µg/ml of CUR does not alter the cell viability *in vitro*. FFA [oleic acid (0.8 mM) and palmitic acid (0.2 mM) could induce significant lipid accumulation in hepatocytes *in vitro*. Pretreatment with GIE, CUR and their combination could significantly reduce the intracellular lipids (Fig. 7A). GIE showed a better effect as the inhibition was slightly higher than CUR, even at a much lower concentration. Curcuminoids are well known for their anti-inflammatory activity, mediated through the inhibition of NFκB. We observed a significant reduction in phosphorylated NFκB in hepatocytes treated with Curcuminoids and the combination of GIE and Curcuminoids (Fig. 7B,C). The ratio of P NFκB to NFκB was also significantly lowered by curcuminoids. GIE was not very effective at the concentration tested. In corroboration with these results, Curcuminoids were highly active in reducing the inflammatory cytokine secretion from monocytes stimulated with bacterial lipopolysaccharide (LPS), while GIE showed a weaker inhibition(Table 2). Since oxidative stress contributes to the progression of NASH and we also observed an improvement in antioxidant markers in mice supplemented with GIE and Curcuminoids, we assessed the levels of SOD and NRF2 in the cells. Interestingly, NRF2 and SOD levels were significantly upregulated in cells treated with the combination, compared to FFA treated control (Fig. 7C,D).

**Table 2:** Cytokine levels in culture supernatant ofactivated THP1 monocytestreated with GIE, CUR and their combination.

Sample	Concentration (pgml)				
	IL12p70	TNF-α	IL10	IL 6	IL 1β
UT	0.73 ± 0.66	1.04 ± 0.91	0.62 ± 0.54	3.95 ± 0.91	1.03 ± 0.64
LPS	1.12 ± 0.58	60.28 ± 0.43	1.42 ± 0.48	658.32 ± 344.27	123.80 ± 0.84
GIE	1.23 ± 0.65	61.78 ± 29.54	1.32 ± 0.39	704.50 ± 360.92	121.81 ± 74.45
CUR	0.80 ± 0.35	37.03 ± 27.27	1.85 ± 0.92	444.49v139.51	108.44 ± 90.83
GIE + CUR	1.11 ± 0.25	48.83 ± 24.85	1.78 ± 0.48	446.36 ± 229.09	144.61 ± 91.64

THP 1 human monocytic cells were stimulated with lipopolysaccharide with GIE (0.125 µg/ml), Curcuminoids (0.625 µg/ml) and their combination (GIE: 0.125 µg/ml+Curcuminoids 0.625 µg/ml) for 24 hours. The supernatants were analyzed for Cytokine levels by Cytokine bead array (CBA array BD biosciences) using flow cytometer.

## Discussion

In the present study, we examined the combinatorial effect of GIE and Curcuminoids in reducing the severity of NASH in an established STAM mouse model. This model offers the advantage of monitoring the liver degeneration from simple fatty liver to NASH and fibrosis. The major advantage of STAM mice is its well-characterized macroscopic and histopathological features, resembling human NASH and fibrosis which has enabled testing of several pharmacological drugs for anti-NASH activity^43^. This model is also recommended for investigating NASH endpoints, including steatosis, inflammation, ballooning, and fibrosis, in a relatively short time frame^44^. This is perhaps the first report of an herbal composition showing efficacy in reducing NAFLD score as well as fibrosis in this complex mouse model. Earlier studies with milk thistle extract had not resulted in a significant decrease in NAS in this model^45^.

The development of NASH is considered as a two-hit model. The first hit being simple steatosis followed by cellular stress and inflammation, giving rise to a fibrotic state, which can lead to hepatocellular carcinoma. Several drugs and antibodies are in different stages of clinical trials for their efficacy in controlling liver fibrosis, while no specific treatment option is still available^46^. In this context, our study provides compelling evidence for the reduction in NAFLD activity score, liver fibrosis, inflammation and related markers in mice supplemented with Curcuminoids and GIE.

The combination ofGIE and Curcuminoids showed a significant decrease in hepatic steatosis and ballooning, resulting in a considerable decrease in NAFLD activity score (NAS), which is one of the clinical endpoints for assessing the activity of NASH^47^. Liver fibrosis is a characteristic feature which defines the prognosis as well as mortality associated with NASH^48,49^. Treatment with the combination showed a significant decrease in liver hydroxyproline content and a decreasing trend in the fibrosis area. Thus, the reduction of hepatocyte ballooning in the GIE + Curcuminoids groups may underlie the anti-fibrosis effects observed in this study.

Despite the decreased steatosis score, blood glucose level and liver triglyceride contents did not differ between treated and control mice. The steatosis score is based on quantitative evaluation of micro- and macro vesicular fat deposition, where triglycerides derived from minuscule lipid droplets and the portal system are not included. Therefore, we speculate that this could be one of the reasons why the decrease in hepatic steatosis and liver triglyceride levels are not correlated in our study.

Myofibroblasts play a vital pathological role in tissue fibrosis by their enhanced ECM production and contractile force generation to contribute to the activation of integrin-bound latent TGF-β1^50^. Fibroblast to myofibroblast trans-differentiation can be induced by transforming  growth factor-β_1_(TGF-β1) by increasing the expression of α-SMA and collagen-1^51^. Myofibroblast increase also correlates with the severity of liver fibrosis in patients^52^,thus, becoming an attractive target for anti-fibrotic therapy^53^. Supplementation with GIE and Curcuminoids combination reduced the TGF-β, collagen I, α-SMA, suggesting they could probably influence the fibroblast-to-myofibroblast transition.

Both lipid and inflammatory components mediate the pathogenesis of NASH. Activation of proinflammatory transcription factor activator protein-1 (AP-1), neutrophil infiltration and NF-*κ*B activation were recently demonstrated to be the leading factors which induce NASH^54^. The chemokine- MCP-1 or CCL2, secreted by macrophages and hepatic stellate cells^55^, regulates fat metabolism and lipid accumulation in hepatocytes and is upregulated in the liver of NASH animals^55–57^. We observed a significant effect of GIE on MCP-1 expression, while Curcuminoids showed a moderate effect. In corroboration, steatosis scores were also reduced by GIE to a greater extent compared to Curcuminoids.

The expression of CD206 and that of Arginase 1 were higher in GIE and GIE + Curcuminoids treated livers, suggesting that GIE possibly modulates the macrophage pool in the liver. Progression from NAFL to NASH is reported to be characterized by the modification of the resident macrophages towards the M1 phenotype by inflammatory mediators, and by the overload of lipids, linking hepatic steatosis to obesity and metabolic syndrome^58,59^. The number of CD206^+^cells, representing M2 macrophages, was reported to lower in paediatric NASH cases^60,61^. Hepatic M2 macrophages play a protective role against liver injury in NAFLD by inducing apoptosis of pathogenic M1 macrophages by an arginase 1 dependent mechanism^62^. Arginase1 expressed by M2 macrophages is a key marker that confers anti-inflammatory properties. The enzyme competes with pro-inflammatory nitric oxide synthetase (iNOS), marker of M1 macrophages, which induces oxidative stress^63^. Curcuminoids were found to reduce inflammatory markers, including TNF-α, C reactive protein and NFkB expression in the liver, while GIE showed only a moderate effect on these markers. Thus,GIE and Curcuminoids may be involved in different signaling mechanisms to manifest their liver protective activity, which warrants further studies.

In the last decade, fibroblast growth factor (FGF)-21 has emerged as an essential regulator of NASH progression^64^. FGF 21 is secreted by metabolically active tissues like the liver, pancreas and muscles and induces insulin sensitivity and energy expenditure^65^. In the liver, FGF21 regulates hepatic glucose production and fatty acid oxidation and prevents diet-induced obesity and hepatic steatosis^66^. Oxidative stress, ER stress, nutritional excess and mitochondrial disorders increase the expression of FGF 21^67,68^. The circulating levels of FGF 21 are elevated in patients with NAFLD/NASH, insulin resistance and obesity^42^, and also in animal models of NASH and obesity, suggesting a state of FGF21 resistance under these conditions^69^. Recently, short term curcumin administration was reported to stimulate FGF21 expression in primary liver fibroblasts in mice. However, the increased hepatic and serum FGF21 in response to HFD was attenuated by curcumin^70^. We observed a reduction in serum and liver FGF21 in the mice treated with curcuminoids alone and in combination with GIE, suggesting that Curcuminoids may act by attenuating HFD induced FGF21 resistance.

Adiponectin is a crucial molecule in metabolic syndrome and is known to prevent progression of steatohepatitis by reducing hepatic inflammation, hepatomegaly, and lipid accumulation and by regulating oxidative stress and Kupffer cell polarization^71,72^. We observed an increase in serum adiponectin levels in mice treated with GIE and Curcuminoids. Interestingly, FGF21 is known to modulate the expression of adiponectin, which in turn plays a significant role in mediating the metabolic effects of FGF21^73^. These results suggest, that by relieving FGF 21 resistance, the combination of GIE and Curcuminoids activate the downstream molecules which are responsible in reducing the metabolic syndrome in the liver

Oxidative stress through lipotoxicity is well known to play an important role in the pathogenesis of NASH. An increase in free fatty acids induces the generation of toxic lipid metabolites, which activate reactive oxygen species (ROS)^74^. Glutathione (GSH) and glutathione peroxidase (GPx) acts as a buffer to protect crucial proteins against pathological modifications and are often known to be depleted in NAFLD^75^. Additionally, Malondialdehyde (MDA) is a product of lipid peroxidation and a sensitive and reliable biomarker of oxidative tissue damage, which has been detected in blood samples from cirrhotic patients^76^. Individual treatment with GIE and Curcuminoids, as well as the combination, could increase the antioxidant levels in the liver to counter the ROS and in turn, reduce the MDA in liver and blood, which further confirms the antioxidant benefits of GIE and C3 Complex. Reduction in oxidative stress induced by FFA was also observed in the HepG2 cells treated with the combination of Curcuminoids and GIE. The NF-E2 p45-related factor 2 (Nrf2) is a master regulator of cellular redox homeostasis^77^. In mice with diet-induced NAFLD, pharmacological activation of NRF2 was reported to inhibit hepatic steatosis, inflammation and fibrosis^78^. Nrf2 influences the changes in gene expression to reduce lipogenesis, ER stress, inflammation, oxidative stress, and fibrosis^78^. In this context, the significant upregulation of NRF2 protein in the cells treated with the combination suggests an additional mechanism of action for the GIE + Curcuminoid combination.

Both Curcuminoids and Garcinol are known to have antioxidant properties, with evidence of liver protection against damage induced by alcohol, drugs and other agents^32,79,80^. Curcuminoids have been subjected to several preclinical and clinical studies for its health benefits related to chronic inflammatory diseases, and Garcinol has been evaluated for its anti-cancer activities and anti-obesity activity by preventing gut dysbiosis which is one of the contributing factors for NAFLD^81^. GIE has been reported to have anti adipogenesis activity and we have also reported an anti-obesity activity of GIE by reducing ER stress and activating adipocyte browning^82^. GIE is thus likely to have a positive effect on reducing steatosis, while curcuminoids being potent anti-inflammatory molecules act on inflammatory pathways. Our results from the *in vitro* studies also confirm this observation. Further experiments *in vitro* with hepatic stellate cells could confirm the exact mechanism of action of these products.

It should be noted that although we observed significant changes in inflammatory markers in the liver, the inflammatory foci did not show a significant difference in the histological sections. One possible explanation for this discrepancy could be that the changes of gene and protein expression level precede histological changes in the STAM model and we might have detected a significant improvement in inflammatory foci on if the mice were treated for a longer duration until 10 weeks or 12 weeks. We also did not observe any change in liver weight, which could be because a reduction of fibrosis seldom affects the liver weight of STAM mice.

In conclusion, our results suggest that while Curcuminoids help in reducing inflammation mediated by NFkB and fibrosis, GIE is effective in modulating macrophage activity and in reducing steatosis. Together the combination could effectively reduce the pathological complications in a well-established and complex animal model of NASH.

NAFLD is one of the most important causes of liver disease worldwide in adults and children, with global prevalence close to 24%^83^. Patients with NAFLD have high-risk metabolic comorbidity, which poses a heavy burden on the health-care systems. The therapeutic strategy in NAFLD is to prevent the progression of liver steatosis to inflammation and fibrosis and to prevent oxidative stress. Thus, a multifaceted approach targeting different aspects is required to treat the disease. The combination of GIE and Curcuminoids is likely to help in attacking these multiple pathways by reducing inflammation, oxidative stress, ER stress and improving gut health to slow down the progression of fatty liver to the more complex stages of NAFLD and NASH. Although the present study was carried out in an established model of NAFLD, supplementation with this combination may be more effective if started at earlier stages of the disease. A clinical trial is underway for further evaluating this combination to further substantiate the potential of GIE + Curcuminoids supplementation in reducing the burden of chronic liver disease.

## Supplementary information

Supplementary information.

## References

[1] Loomba R, Sanyal AJ (2013). The global NAFLD epidemic. Nat. Rev. Gastroenterol. Hepatol..

[2] Lazo M (2013). Prevalence of nonalcoholic fatty liver disease in the United States: the Third National Health and Nutrition Examination Survey, 1988-1994. Am. J. Epidemiol..

[3] Chalasani N (2012). The diagnosis and management of non-alcoholic fatty liver disease: practice Guideline by the American Association for the Study of Liver Diseases, American College of Gastroenterology, and the American Gastroenterological Association. Hepatology.

[4] Williams CD (2011). Prevalence of nonalcoholic fatty liver disease and nonalcoholic steatohepatitis among a largely middle-aged population utilizing ultrasound and liver biopsy: a prospective study. Gastroenterology.

[5] Charlton MR (2011). Frequency and outcomes of liver transplantation for nonalcoholic steatohepatitis in the United States. Gastroenterology.

[6] Sanyal A, Poklepovic A, Moyneur E, Barghout V (2010). Population-based risk factors and resource utilization for HCC: US perspective. Curr. Med. Res. Opin..

[7] Ratziu V, Bellentani S, Cortez-Pinto H, Day C, Marchesini G (2010). A position statement on NAFLD/NASH based on the EASL 2009 special conference. J. Hepatol..

[8] Yki-Jarvinen H (2014). Non-alcoholic fatty liver disease as a cause and a consequence of metabolic syndrome. Lancet Diabetes Endocrinol..

[9] Angulo P (2015). Liver Fibrosis, but No Other Histologic Features, Is Associated With Long-term Outcomes of Patients With Nonalcoholic Fatty Liver Disease. Gastroenterology.

[10] Ekstedt M (2015). Fibrosis stage is the strongest predictor for disease-specific mortality in NAFLD after up to 33 years of follow-up. Hepatology.

[11] Tilg H, Moschen AR (2010). Evolution of inflammation in nonalcoholic fatty liver disease: the multiple parallel hits hypothesis. Hepatology.

[12] Marra F, Lotersztajn S (2013). Pathophysiology of NASH: perspectives for a targeted treatment. Curr. Pharm. Des..

[13] Rinella ME (2015). Nonalcoholic fatty liver disease: a systematic review. JAMA.

[14] Fujii H, Kawada N (2012). Inflammation and fibrogenesis in steatohepatitis. J. Gastroenterol..

[15] Zhang XQ, Xu CF, Yu CH, Chen WX, Li YM (2014). Role of endoplasmic reticulum stress in the pathogenesis of nonalcoholic fatty liver disease. World J. Gastroenterol..

[16] Puri P (2008). Activation and dysregulation of the unfolded protein response in nonalcoholic fatty liver disease. Gastroenterology.

[17] Rinella ME (2011). Dysregulation of the unfolded protein response in db/db mice with diet-induced steatohepatitis. Hepatology.

[18] Gregor MF (2009). Endoplasmic reticulum stress is reduced in tissues of obese subjects after weight loss. Diabetes.

[19] Ozcan U (2004). Endoplasmic reticulum stress links obesity, insulin action, and type 2 diabetes. Science.

[20] Rahman SM (2007). CCAAT/enhancing binding protein beta deletion in mice attenuates inflammation, endoplasmic reticulum stress, and lipid accumulation in diet-induced nonalcoholic steatohepatitis. Hepatology.

[21] Wang D, Wei Y, Pagliassotti MJ (2006). Saturated fatty acids promote endoplasmic reticulum stress and liver injury in rats with hepatic steatosis. Endocrinology.

[22] Dyson, J. K., Anstee, Q. M.&McPherson, S.Non-alcoholic fatty liver disease: a practical approach to treatment. Frontline Gastroenterology, 10.1136/flgastro-2013-100404 (2014). 10.1136/flgastro-2013-100404 PMC4173737 25285192

[23] Ratziu V (2016). Elafibranor, an Agonist of the Peroxisome Proliferator-Activated Receptor-alpha and -delta, Induces Resolution of Nonalcoholic Steatohepatitis Without Fibrosis Worsening. Gastroenterology.

[24] Epstein J, Sanderson IR, Macdonald TT (2010). Curcumin as a therapeutic agent: the evidence from *in vitro*, animal and human studies. Br. J. Nutr..

[25] Afrin R (2017). Curcumin ameliorates liver damage and progression of NASH in NASH-HCC mouse model possibly by modulating HMGB1-NF-kappaB translocation. Int. Immunopharmacol..

[26] Wang L, Lv Y, Yao H, Yin L, Shang J (2015). Curcumin prevents the non-alcoholic fatty hepatitis via mitochondria protection and apoptosis reduction. Int. J. Clin. Exp. Pathol..

[27] Soliman MM, Abdo Nassan M, Ismail TA (2014). Immunohistochemical and molecular study on the protective effect of curcumin against hepatic toxicity induced by paracetamol in Wistar rats. BMC Complement. Altern. Med..

[28] Fu Y, Zheng S, Lin J, Ryerse J, Chen A (2008). Curcumin protects the rat liver from CCl4-caused injury and fibrogenesis by attenuating oxidative stress and suppressing inflammation. Mol. Pharmacol..

[29] Yamaguchi F, Ariga T, Yoshimura Y, Nakazawa H (2000). Antioxidative and anti-glycation activity of garcinol from Garcinia indica fruit rind. J. Agric. Food Chem..

[30] Pan MH, Chang WL, Lin-Shiau SY, Ho CT, Lin JK (2001). Induction of apoptosis by garcinol and curcumin through cytochrome c release and activation of caspases in human leukemia HL-60 cells. J. Agric. Food Chem..

[31] Balasubramanyam K (2004). Polyisoprenylated benzophenone, garcinol, a natural histone acetyltransferase inhibitor, represses chromatin transcription and alters global gene expression. J. Biol. Chem..

[32] Hung WL (2014). Protective effects of garcinol on dimethylnitrosamine-induced liver fibrosis in rats. Food Funct..

[33] Fujii M (2013). A murine model for non-alcoholic steatohepatitis showing evidence of association between diabetes and hepatocellular carcinoma. Med. Mol. Morphol..

[34] Yoshioka T, Kawada K, Shimada T, Mori M (1979). Lipid peroxidation in maternal and cord blood and protective mechanism against activated-oxygen toxicity in the blood. Am. J. Obstet. Gynecol..

[35] Folch J, Lees M, Sloane Stanley GH (1957). A simple method for the isolation and purification of total lipides from animal tissues. J. Biol. Chem..

[36] Buege JA, Aust SD (1978). Microsomal lipid peroxidation. Methods Enzymol..

[37] Hafeman DG, Sunde RA, Hoekstra WG (1974). Effect of dietary selenium on erythrocyte and liver glutathione peroxidase in the rat. J. Nutr..

[38] Moron MS, Depierre JW, Mannervik B (1979). Levels of glutathione, glutathione reductase and glutathione S-transferase activities in rat lung and liver. Biochim. Biophys. Acta.

[39] Kleiner DE (2005). Design and validation of a histological scoring system for nonalcoholic fatty liver disease. Hepatology.

[40] Lin Y (2017). Downregulation of miR-192 causes hepatic steatosis and lipid accumulation by inducing SREBF1: Novel mechanism for bisphenol A-triggered non-alcoholic fatty liver disease. Biochim. Biophys. Acta Mol. Cell Biol. Lipids.

[41] Dushay J (2010). Increased fibroblast growth factor 21 in obesity and nonalcoholic fatty liver disease. Gastroenterology.

[42] Barb D, Bril F, Kalavalapalli S, Cusi K (2019). Plasma Fibroblast Growth Factor 21 Is Associated With Severity of Nonalcoholic Steatohepatitis in Patients With Obesity and Type 2 Diabetes. J. Clin. Endocrinol. Metab..

[43] Hansen HH (2017). Mouse models of nonalcoholic steatohepatitis in preclinical drug development. Drug. Discov. Today.

[44] Cole BK, Feaver RE, Wamhoff BR, Dash A (2018). Non-alcoholic fatty liver disease (NAFLD) models in drug discovery. Expert. Opin. Drug. Discov..

[45] Pais P, D’Amato M (2014). *In vivo* efficacy study of milk thistle extract (ETHIS-094) in STAM model of nonalcoholic steatohepatitis. D.rugs R. D..

[46] Fagone P (2016). Emerging therapeutic targets for the treatment of hepatic fibrosis. Drug. Discovery Today.

[47] Sanyal AJ (2011). Endpoints and clinical trial design for nonalcoholic steatohepatitis. Hepatology.

[48] Singh, S.et al.Fibrosis progression in nonalcoholic fatty liver vs nonalcoholic steatohepatitis: a systematic review and meta-analysis of paired-biopsy studies. Clin Gastroenterol Hepatol 13, 643-654 e641-649; quize639-640, 10.1016/j.cgh.2014.04.014 (2015). 10.1016/j.cgh.2014.04.014 PMC4208976 24768810

[49] Chalasani N (2018). The diagnosis and management of nonalcoholic fatty liver disease: Practice guidance from the American Association for the Study of Liver Diseases. Hepatology.

[50] Hinz B (2012). Recent developments in myofibroblast biology: paradigms for connective tissue remodeling. Am. J. Pathol..

[51] Zhang K, Rekhter MD, Gordon D, Phan SH (1994). Myofibroblasts and their role in lung collagen gene expression during pulmonary fibrosis. A combined immunohistochemical and *in situ* hybridization study. Am. J. Pathol..

[52] Brenner DA (2012). Origin of myofibroblasts in liver fibrosis. Fibrogenesis Tissue Repair..

[53] Kisseleva T (2012). Myofibroblasts revert to an inactive phenotype during regression of liver fibrosis. Proc. Natl Acad. Sci. USA.

[54] Liang, W.et al.Metabolically induced liver inflammation leads to NASH and differs from LPS- or IL-1β-induced chronic inflammation. Laboratory Investigation94, 491, 10.1038/labinvest.2014.11https://www.nature.com/articles/labinvest201411#supplementary-information (2014). 10.1038/labinvest.2014.11 24566933

[55] Wobser H (2009). Lipid accumulation in hepatocytes induces fibrogenic activation of hepatic stellate cells. Cell Res..

[56] Rull A (2009). Hepatic monocyte chemoattractant protein-1 is upregulated by dietary cholesterol and contributes to liver steatosis. Cytokine.

[57] Obstfeld AE (2010). C-C chemokine receptor 2 (CCR2) regulates the hepatic recruitment of myeloid cells that promote obesity-induced hepatic steatosis. Diabetes.

[58] Eckert C, Klein N, Kornek M, Lukacs-Kornek V (2015). The complex myeloid network of the liver with diverse functional capacity at steady state and in inflammation. Front. Immunol..

[59] Seki E, Schwabe RF (2015). Hepatic inflammation and fibrosis: functional links and key pathways. Hepatology.

[60] Carpino G (2016). Macrophage Activation in Pediatric Nonalcoholic Fatty Liver Disease (NAFLD) Correlates with Hepatic Progenitor Cell Response via Wnt3a Pathway. PLoS One.

[61] de Oliveira, F. L.et al.The Number of Liver Galectin-3 Positive Cells Is Dually Correlated with NAFLD Severity in Children. Int J Mol Sci20, 10.3390/ijms20143460 (2019). 10.3390/ijms20143460 PMC6679049 31337151

[62] Wan J (2014). M2 Kupffer cells promote M1 Kupffer cell apoptosis: a protective mechanism against alcoholic and nonalcoholic fatty liver disease. Hepatology.

[63] Scheja L, Kluwe J (2015). Arginine and NASH–do macrophages deliver the first hit?. J. Hepatol..

[64] Fisher FM, Maratos-Flier E (2016). Understanding the Physiology of FGF21. Annu. Rev. Physiol..

[65] Gimeno RE, Moller DE (2014). FGF21-based pharmacotherapy–potential utility for metabolic disorders. Trends Endocrinol. Metab..

[66] Xu J (2009). Fibroblast growth factor 21 reverses hepatic steatosis, increases energy expenditure, and improves insulin sensitivity in diet-induced obese mice. Diabetes.

[67] Liu Y (2012). Cobalt chloride decreases fibroblast growth factor-21 expression dependent on oxidative stress but not hypoxia-inducible factor in Caco-2 cells. Toxicol. Appl. Pharmacology.

[68] Schaap FG, Kremer AE, Lamers WH, Jansen PL, Gaemers IC (2013). Fibroblast growth factor 21 is induced by endoplasmic reticulum stress. Biochimie.

[69] Liu J, Xu Y, Hu Y, Wang G (2015). The role of fibroblast growth factor 21 in the pathogenesis of non-alcoholic fatty liver disease and implications for therapy. Metab. - Clin. Exp..

[70] Zeng K (2017). Diet Polyphenol Curcumin Stimulates Hepatic Fgf21 Production and Restores Its Sensitivity in High-Fat-Diet-Fed Male Mice. Endocrinology.

[71] Rogers CQ, Ajmo JM, You M (2008). Adiponectin and alcoholic fatty liver disease. IUBMB Life.

[72] Fukushima J (2009). Adiponectin prevents progression of steatohepatitis in mice by regulating oxidative stress and Kupffer cell phenotype polarization. Hepatol. Res..

[73] Lin Z (2013). Adiponectin Mediates the Metabolic Effects of FGF21 on Glucose Homeostasis and Insulin Sensitivity in Mice. Cell Metab..

[74] Neuschwander-Tetri BA (2010). Hepatic lipotoxicity and the pathogenesis of nonalcoholic steatohepatitis: the central role of nontriglyceride fatty acid metabolites. Hepatology.

[75] Oz HS, Im HJ, Chen TS, de Villiers WJ, McClain CJ (2006). Glutathione-enhancing agents protect against steatohepatitis in a dietary model. J. Biochem. Mol. Toxicol..

[76] Zheng W (2019). Increased serum malondialdehyde levels are associated with grey matter volume loss in patients with non-alcoholic cirrhosis. Quant. Imaging Med. Surg..

[77] Hayes JD, Dinkova-Kostova AT (2014). The Nrf2 regulatory network provides an interface between redox and intermediary metabolism. Trends Biochem. Sci..

[78] Sharma RS (2018). Experimental Nonalcoholic Steatohepatitis and Liver Fibrosis Are Ameliorated by Pharmacologic Activation of Nrf2 (NF-E2 p45-Related Factor 2). Cell Mol. Gastroenterol. Hepatol..

[79] Rivera-Espinoza Y (2009). & Muriel, P. Pharmacological actions of curcumin in liver diseases or damage. Liver Int..

[80] Lee HY (2017). Curcumin and Curcuma longa L. extract ameliorate lipid accumulation through the regulation of the endoplasmic reticulum redox and ER stress. Sci. Rep..

[81] Leung C, Rivera L, Furness JB, Angus PW (2016). The role of the gut microbiota in NAFLD. Nat. Rev. Gastroenterol. Hepatol..

[82] Majeed M, Majeed S, Nagabhushanam K, Lawrence L, Mundkur L (2020). Garcinia indica extract standardized for 20% Garcinol reduces adipogenesis and high fat diet-induced obesity in mice by alleviating endoplasmic reticulum stress. J. Funct. Foods.

[83] Younossi Z (2018). Global burden of NAFLD and NASH: trends, predictions, risk factors and prevention. Nat. Rev. Gastroenterol. Hepatol..

