# Supplementary material for: Novel Combinatorial Regimen of Garcinol and Curcuminoids for Non-alcoholic Steatohepatitis (NASH) in Mice
Source: Sci Rep. 2020 May 4;10:7440. doi: 10.1038/s41598-020-64293-w (PMC7198554; doi:10.1038/s41598-020-64293-w)

**Novel Combinatorial Regimen of Garcinol and Curcuminoids for Non-alcoholic Steatohepatitis (NASH) in Mice**

Muhammed Majeed^1, 2, 3^ Shaheen Majeed ^2^, Kalyanam Nagabhushanam^3^ Lincy Lawrence^1^, Lakshmi Mundkur^1*^

^1^Sami Labs Limited, Peenya Industrial Area, Bangalore 560 058, Karnataka, India.

^2^Sabinsa Corporation, 750 Innovation Circle, Payson, UT 84651, USA.

^3^Sabinsa Corporation, 20 Lake Drive, East Windsor, NJ 08520, USA

***Corresponding author**

Dr. Lakshmi Mundkur

Biological R&D

Sami labs Ltd

19/1, 19/2, 1st Main, 2nd Phase,

Peenya Industrial Area,

Bengaluru, Karnataka 560058

Tel 080 2839 7973

lakshmi@samilabs.com

**Short Title-**

Synergy of Garcinol and Curcuminoids in NASH

**Author Contributions**

Experimentation and data collection; LL, LM Data Validation: LM, Resources: MM and SM. Writing (original draft): LL and LM. Writing (review & editing): KN, SM, LM Approval: SM, KN, and MM

**Methods**

**Details of guidelines followed for animal experiments**

All the methods using animals were carried out in accordance with relevant guidelines and regulations with approval from the animal ethics committee of SMC laboratories Japan (IACUC.). The laboratory has the approval to conduct in-vivo studies as per the Animal Welfare Assurance for foreign institutions from the Office of Laboratory Animal Welfare on November 30, 2015 (Animal Welfare Assurance number: A5037-01) as per the following guidelines

1) Act on Welfare and Management of Animals (Ministry of the Environment, Act No. 105 of October 1, 1973)

2) Standards Relating to the Care and Management of Laboratory Animals and Relief of Pain (Notice No.88 of the Ministry of the Environment, April 28, 2006)

3) Guidelines for Proper Conduct of Animal Experiments (Science Council of Japan, June 1, 2006)

The animals and monitored for viability, clinical signs (lethargy, twitching, labored breathing) and behavior daily. Mice were observed for significant clinical signs of toxicity, moribundity and mortality before administration, just after administration and 1 hours after administration. The animals were sacrificed by exsanguination through direct cardiac puncture under isoflurane anesthesia (Pfizer Inc.).

If an animal showed >25% body weight loss within a week or >20% body weight loss compared to previous day, the animal was euthanized ahead of study termination. If it showed a morbidity sign such as prone position, the animals were euthanized ahead of study termination.

**Measurement of liver hydroxyproline content**

Frozen liver samples were processed by an alkaline-acid hydrolysis method to quantify the liver hydroxyproline content. Liver samples were defatted with 100% acetone, dried in the air, dissolved in 2N NaOH at 65°C, and autoclaved at 121°C for 20 minutes. The lysed samples (400 µL) were acid-hydrolyzed with 400 µL of 6N HCl at 121°C for 20 minutes and neutralized with 400 µL of 4N NaOH containing 10 mg/mL activated carbon. AC buffer (2.2M acetic acid/0.48M citric acid, 400 µL) was added to the samples, followed by centrifugation to collect the supernatant. A standard curve of hydroxyproline was constructed with serial dilutions of trans-4-hydroxy-L-proline (Sigma-Aldrich) starting at 16 µg/mL. The prepared samples and standards (each 400 µL) were mixed with 400 µL chloramine T solution (Wako Pure Chemical Industries) and incubated for 25 minutes at room temperature. The samples were then mixed with Ehrlich's solution (400 µL) and heated at 65°C for 20 minutes to develop the color. After samples were cooled on ice and centrifuged to remove precipitates, the optical density of each supernatant was measured at 560 nm. The concentrations of hydroxyproline were calculated from the hydroxyproline standard curve. Protein concentrations of liver samples were determined using a BCA protein assay kit (Thermo Fisher Scientific, USA) and used to normalize the calculated hydroxyproline values and expressed as µg per mg protein.

**Glutathione peroxidase (GPx)**

Glutathione peroxidase activity was determined according to the method of Hafeman, et al. ^37^. Glutathione peroxidase degrades H_2_O_2_ in the presence of glutathione (GSH), thereby depleting it. The remaining GSH is measured using Ellman's reagent (5,5′-dithio-bis(2-nitrobenzoic acid) (DTNB), which gives a colored complex. To a volume of 100 μl of cell lysate, 0.2 mM GSH, 0.05 mM H_2_O_2_ and 1 mM NaN_3_ were added and the volume was made up to 250 µl with 1M sodium phosphate buffer (pH 7.0). The reaction was incubated at 37ºC for 10 min and stopped by adding 50 µl of 25% TCA. The reaction mixture was centrifuged at 3000 rpm for 10 min and to 0.1 ml of the supernatant, 0.1 ml of 0.4 M Na_2_HPO_4_ and 50 µl of 1 mM DTNB was added. The intensity of yellow color formation was measured at 412 nm after incubation at 37^º^C for 10 min. The enzyme activity was expressed as units/mg protein.

## RNA extraction and quantitative RT-PCR

For cDNA samples, the other 2 pieces of left lateral lobe were snap-frozen in liquid nitrogen and stored at -80°C until use. Total RNA was extracted from liver samples using RNA iso (Takara Bio, Japan) according to the manufacturer's instructions. One μg of RNA was reverse-transcribed using a reaction mixture containing 4.4 mM MgCl_2_ (F. Hoffmann-La Roche, Switzerland), 40 U RNase inhibitor (Toyobo, Japan), 0.5 mM dNTP (Promega, USA), 6.28 μM random hexamer (Promega),5 x first-strand buffer (Promega), 10 mM dithiothreitol (Invitrogen, USA) and 200 U MMLV-RT (Invitrogen) in a final volume of 20 μL. The reaction was carried out for 1 hour at 37°C, followed by 5 minutes at 99°C. The samples were frozen in liquid nitrogen and stored at -80°C until use. Quantitative real-time PCR (qRT-PCR) was performed with SYBR Green I fluorescent dye using Light cycler 96 according to the manufacturer’s instructions (Light Cycler^®^ FastStart DNA Master SYBR Green I, Roche). The primers used for the analysis are provided in Supplementary Table 1. Expression levels for all genes were normalized to β-actin gene amplification. The gene expression of the target gene in each test sample was determined by relative quantification using the comparative Ct (ΔΔCt) method.

**Table 1 List of Primers**

| **Si No** | **Oligo Name** | **Sequence 5’ to 3’** | **Length** |
| --- | --- | --- | --- |
| 1 | m TNα F | CACAGAAAGCATGATCCGCGACGT | 24 |
|  | m TNα R | CGGCAGAGAGGAGGTTGACTTTCT | 24 |
| 2 | m NFκB F | GAAATTCCTGATCCAGACAAAAAC | 24 |
|  | m NFκB R | ATCACTTCAATGGCCTCTGTGTAG | 24 |
| 3 | m MCP-1 F | GCATCCACGTGTTGGCTCA | 19 |
|  | m MCP-1 R | CTCCAGCCTACTCATTGGGATCA | 23 |
| 4 | m CRP F | TGGATTGATGGGAAACCCAA | 20 |
|  | m CRP R | GCATCTGGCCCCACAGTG | 18 |
| 5 | m Arginase I F | TGGCTTTAACCTTGGCTTGCTTCG | 24 |
|  | m Arginase I R | AAAGAACAAGCCCTTGGGAGGAGA | 24 |
| 6 | m Cola1 F | TTCCCTGGACCTAAGGGTACT | 21 |
|  | m Cola1 R | TTGAGCTCCAGCTTCGCC | 18 |
| 7 | m TGFβ F | GACCGCAACAACGCCATCTA | 20 |
|  | m TGFβ R | GGCGTATCAGTGGGGGTCAG | 20 |

**Immunoblotting**

Frozen liver from the animals were homogenized and the cells were lysed using ice-cold RIPA buffer containing protease (1x protease inhibitor cocktail – HI media) and phosphatase (sodium orthovanadate 1 mM) and incubated with constant agitation for 2 h at 4°C. The lysate was centrifuged for 20 min at 12,000 rpm at 4°C and the supernatant was transferred to a fresh sterile tube and stored at -80 °C in small aliquots. Protein concentration was estimated by the Bradford method **(**Sigma, USA). Cellular protein (100 μg) was loaded per lane in denatured 10% polyacrylamide gel (SDS-PAGE). The separated proteins were transferred to a polyvinylidene difluoride membrane (Invitrolon™ PVDF, Thermo Fisher Scientific, USA) and blocked in 5% nonfat dry milk for 2 hours. Membranes were then incubated with the appropriate dilutions of anti-mouse primary antibodies at 4ᵒC for 18 hours, followed by horseradish peroxidase-conjugated secondary antibody (Thermo Scientific, USA) for 2 hours at 37ᵒC. The details of the antibodies are provided in Supplementary Table 2. Immunoreactive protein bands were detected by ECL ((Pierce ECL plus, Thermo Scientific, USA). Immunoblots were quantified using Image J software (version 1.52a, National Institute of Health, USA).

**Table 2 List of Antibodies**

| **Antibody** | **Code** | **Source** |
| --- | --- | --- |
| NFE2L2 Antibody 100ug/ml (NRF2) | CSB-PA003481 | Cusabio |
| RELA (NFKB) | CSB-PA03987A0RB | CUSABIO |
| Phospho - RELA  (S536) (NFKB) | CSB-PA000586 | CUSABIO |
| Anti-FGF21 antibody - 100 ul | AB171941 | Abcam |
| Beta Actin | 4967 | Cell signalling |

**Results**

**Table 3 – Details of calculating NAFLD activity score**

**Figure 1 Full-length western blots**


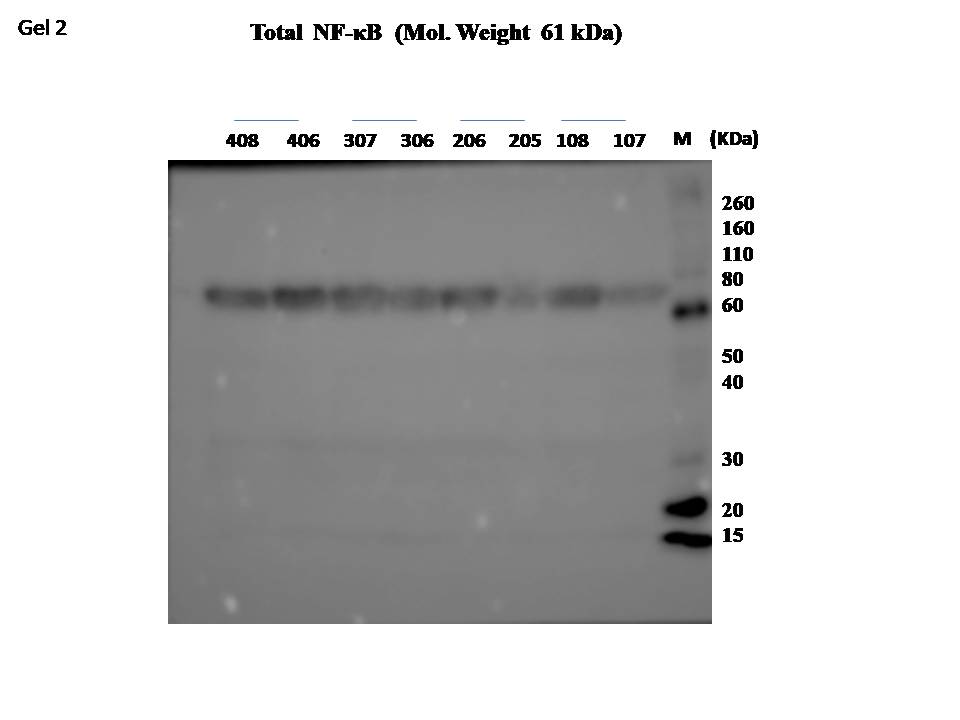


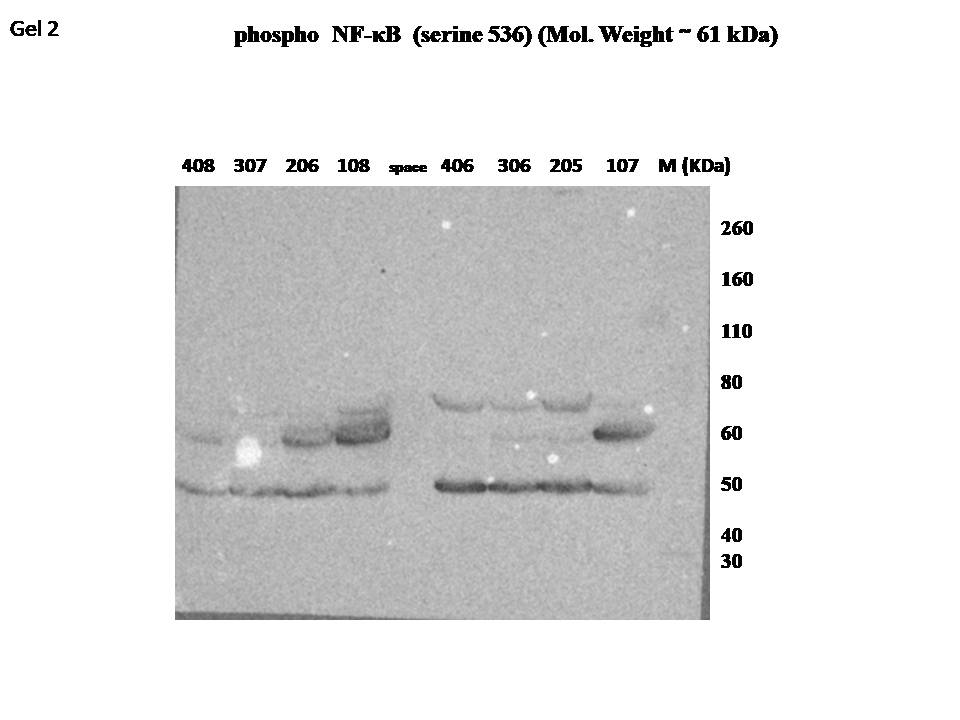

Supplement: Supplementary file 1 — Supplementary information. [file 41598_2020_64293_MOESM1_ESM.docx]
